# Analysis of Types of Skin Lesions and Diseases in Everyday Infectious Disease Practice—How Experienced Are We?

**DOI:** 10.3390/life12070978

**Published:** 2022-06-29

**Authors:** Tomislava Skuhala, Vladimir Trkulja, Marin Rimac, Anja Dragobratović, Boško Desnica

**Affiliations:** 1 University Hospital for Infectious Diseases “Dr. Fran Mihaljević”, 10000 Zagreb, Croatia; adragobratovic@bfm.hr (A.D.); bdesnica@bfm.hr (B.D.); 2 School of Dental Medicine, University of Zagreb, 10000 Zagreb, Croatia; 3 Department of Pharmacology, School of Medicine, University of Zagreb, 10000 Zagreb, Croatia; vladimir.trkulja@mef.hr; 4 School of Medicine, University of Zagreb, 10000 Zagreb, Croatia; mrimac@mef.hr

**Keywords:** skin lesions, infectious, noninfectious, undiagnosed

## Abstract

Rashes and skin lesions are a common reason for patient visits to emergency departments and physicians’ offices. The differential diagnosis includes a variety of infectious and non-infectious diseases, some of which can be life-threatening. The aim of this retrospective study was to evaluate the quantity and type of skin lesions among outpatients and inpatients at a tertiary care university-affiliated teaching hospital for infectious diseases over a three-year period to assess disease burden and physicians’ experience in diagnosing skin lesions. Diagnoses (by ICD-10 codes) were classified into three groups: infectious diseases that include skin lesions, non-infectious skin lesions and undiagnosed skin lesions. During the observed period, out of the total of 142,416 outpatients, 14.8% presented with some form of skin lesion. Among them, 68% had skin lesions inherent to infectious disease, 10.8% suffered from non-infectious skin lesions and 21.2% remained with undiagnosed skin lesions. The most common infectious diagnoses were chickenpox, herpes zoster and unspecified viral infections characterized by skin and mucous membrane lesions. The most common non-infectious diagnoses were urticaria and atopic dermatitis. Overall, the most common individual diagnosis (ICD-10 code) was “nonspecific skin eruption” (*n* = 4448, 21.1%), which was followed by chickenpox and herpes zoster. Among the 17,401 patients hospitalized over the observed period, 13.1% had skin lesion as the main reason for hospitalization, almost all (97.5%) of which were infectious in etiology. The most common diagnoses were cellulitis, erysipelas and herpes zoster. The presented data suggest that the burden of diseases presenting with skin lesions is significant in everyday infectious disease practice, but the overwhelming number of undiagnosed patients implies the need for further education in this area.

## 1. Introduction

Rashes and skin lesions are common reasons for seeking medical services. Generally, skin diseases are among the most frequently occurring illnesses in humans. Skin and subcutaneous disorders were the fourth leading cause of nonfatal disease burden worldwide in the last decade [1,2,3] with increasing prevalence [4]. In 2013, skin conditions contributed 1.79% to the total international burden of disease measured in disability-adjusted life expectancy across 306 different diseases and injuries. An important part of those conditions refers to certain skin conditions caused by infectious agents, such as cellulitis, pyoderma, scabies, and various other fungal and viral skin diseases [2].

The most predominant types of skin lesions in everyday infectious disease practice are skin and soft tissue infections caused by bacteria, viruses, fungi and parasites, and different types of exanthems presenting with fever [5,6,7,8]. Skin and soft tissue infections are among the most widespread types of infections and include cellulitis, erysipelas, impetigo, ecthyma, folliculitis, furuncles, carbuncles, abscesses, trauma-related infections, decubital ulcers, necrotizing fasciitis, Fournier gangrene and infections from human or animal bites. Their presentation, etiology, and severity are variable. The incidence of these infections, which are a common reason for hospitalizations, has increased in the previous decades by up to 65% [9,10,11,12].

In the pediatric population, a combination of characteristic rash and other symptoms is sufficient for the diagnosis of classical exanthems such as measles, scarlet fever, rubella, erythema infectiosum, exanthema subitum and chickenpox [13,14,15]. There are also “atypical exanthems” that may be associated with fever or other constitutional symptoms and may be caused by infectious or toxic agents through hypersensitivity reactions [15,16,17]. Some of them are a diagnostic challenge, but the ones present in medical emergencies and those that pose an urgent diagnostic and therapeutic dilemma are especially important [18].

Differential diagnosis of all the aforementioned conditions is extensive and includes a variety of infectious and non-infectious causes. In some cases, skin lesions are a prominent symptom of systemic or localized disease and are ample for diagnosing certain infections or non-infectious conditions [19,20,21], but a variety of infectious and non-infectious diseases can produce similar skin lesions, and a single condition can present with different types of skin lesions due to a limited number of skin manifestations [6,19,20,21].

The pathogenesis of skin lesions associated with infectious diseases can be variable: (a) direct inoculation and multiplication of microorganisms within the skin, (b) dissemination of microorganisms from a different site, (c) production of toxins that can directly affect skin structure, (d) vascular effects of toxins, (e) inflammatory/immune response to microorganisms or toxins, (f) disseminated intravascular coagulation and coagulopathy, (g) direct vascular invasion and occlusion by microorganisms, and (h) reaction to medications used to treat infectious diseases [10,17,22,23]. The pathogenesis in other, non-infectious skin lesions is even more complex. However, knowing possible mechanisms in the development of skin lesions is not a direct clue in establishing an etiological diagnosis.

Even though a detailed physical examination is crucial, a thorough medical history is also essential where a number of factors must be taken into account in establishing the diagnosis of skin lesions. These include the appearance of skin lesions together with other symptoms, distribution of skin changes, accompanying pruritus, potential exposure to infectious agents, occurrence in family members and colleagues, drug intake, immunization, seasonal occurrence, history of allergy, immune status of the host, contact with animals and exposure to sexually transmitted diseases [6,20,21].

Some skin lesions, such as herpes zoster, psoriasis and atopic dermatitis can be correctly recognized even without significant experience, but many others are more demanding in this respect [24,25]. Training and clinical exposure to dermatological conditions leads to a greater ability to establish the correct diagnosis not only by dermatologists but also by other physicians. Infectious skin diseases are among the most common dermatologic conditions reported by non-dermatologists [25,26]. In certain diseases, skin lesions can be an early sign of life-threatening infections (meningococcal infection, toxic shock syndrome, necrotizing fasciitis, staphylococcal scalded skin syndrome) or non-infectious diseases (toxic epidermal necrolysis, Steven–Johnson syndrome, angio-edema). Rapid diagnosis is essential in order to start timely treatment. Some skin lesions are present during diseases that must be treated to prevent serious consequences (syphilis, cellulitis, scarlet fever, varicella in adults, exanthema chronicum migraines, etc.), and some skin lesions are part of self-limited diseases (exanthema subitum, enteroviral infections, erythema infectiosum). It is crucial for clinicians to have a basic knowledge of infectious diseases while dealing with skin lesions, but a comprehensive knowledge of other non-infectious diseases is also required. Experience and appropriate education in these areas are critically important and can be lifesaving.

We aimed to assess disease burden and physicians’ experience in diagnosing skin lesions in outpatients and inpatients at a tertiary care university-affiliated teaching hospital for infectious diseases.

## 2. Patients and Methods

We retrospectively searched institutional administrative data collected between 1 January 2015 and 31 December 2017 on all outpatient visits and hospitalizations at a tertiary care university-affiliated teaching hospital in order to identify the International Classification of Disease version 10 (ICD-10, 2015) codes assigned to: (a) infectious diseases in which rash/skin lesions are a major element of clinical presentation; (b) infectious diseases in which rash/skin lesions are a common element of clinical presentation; and (c) rash/skin lesions unrelated to infectious diseases classified in the chapter “Diseases of the skin and subcutaneous tissues” (L00-L99) of the ICD-10—if they were the main reason for the patient’s visit/hospitalization. The searched ICD-10 codes are listed in Table 1 and Table 2. We disregarded: (a) infectious diseases in which rash/skin lesions may occasionally be present; and (b) diseases classified as ICD-10 codes L00-L99, in which skin is typically not affected, even if they were the main reason (leading diagnosis) for the patient’s visit. All included patients were first examined in our emergency department specifically organized for patients with confirmed or suspected infectious diseases. The patients who were diagnosed and treated there were considered outpatients, while those who had to be hospitalized were considered inpatients. We used routinely collected anonymized data; hence, the need for ethical approval was waived by the Institutional Ethics Committee.

## 3. Results

### 3.1. Outpatients

**Figure 1:**
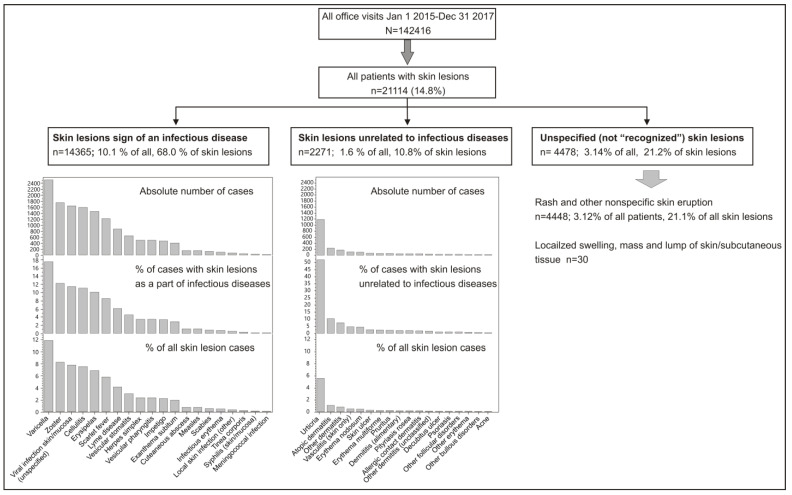
Breakdown of outpatient visits included in the present analysis.

Of the 142,416 outpatient visits recorded during the observed period, 21,114 (14.8%) presented with some form of a skin lesion (Figure 1). Among them, 14,365 patients (68.0%) presented with skin lesions inherent to an infectious disease, 2271 (10.8%) were deemed to have suffered from non-infectious skin lesions and 4478 patients (21.2% of managed patients with skin lesions) remained with undiagnosed skin lesions (Figure 1). The overall most common individual ICD-10 code (diagnosis) was “rash and other nonspecific skin eruption” or, in other words, not recognized skin lesions—in 4448 patients (21.1% of those who presented with skin lesions and 3.1% of all outpatient visits) (Figure 1). The other most common diagnoses pertaining to infectious diseases included: chickenpox (*n* = 2513, 11.9% of all skin lesions), herpes zoster (*n* = 1754, 8.3%), unspecified viral infections characterized by skin and mucous membrane lesions (*n* = 1646, 7.8%), cellulitis (*n* = 1590, 7.5%), erysipelas (*n* = 1451, 6.9%), scarlet fever (*n* = 1223, 5.8%) and Lyme disease (*n* = 876, 4.1%) (Figure 1). The most common non-infectious diagnoses were urticaria (*n* = 1180, 5.6%), atopic dermatitis (*n* = 232, 1.1%) and “other” dermatitis (*n* = 168, 0.8%) (Figure 1).

### 3.2. Inpatients

**Figure 2:**
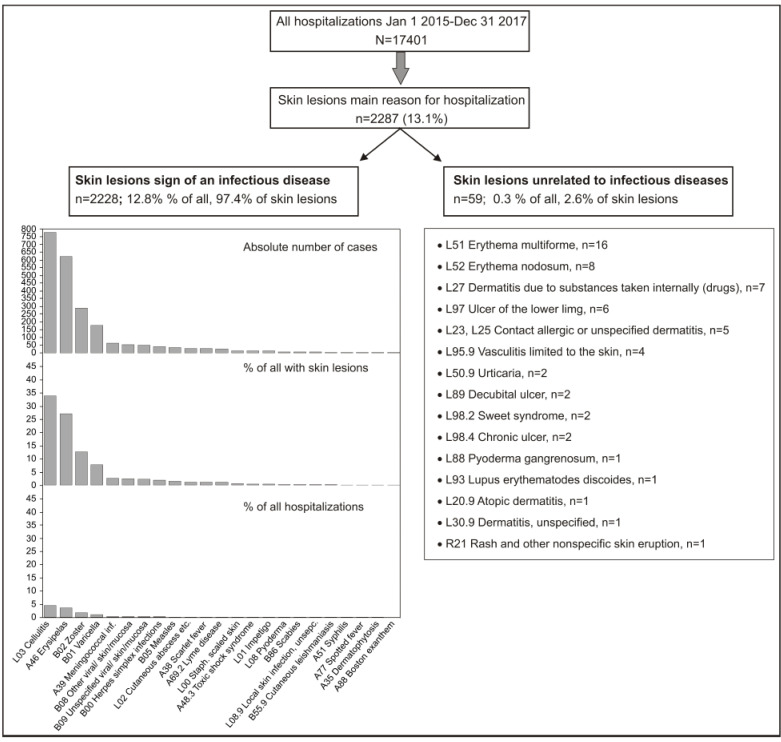
Breakdown of hospitalized patients included in the present analysis.

Of the 17,401 patients hospitalized over the observed period, 2287 (13.1%) had skin lesions as the main reason for hospitalization (Figure 2): almost all (*n* = 2228; 97.4% of skin lesions, 12.8% of all hospitalizations) were infectious in etiology; in only 59 patients (2.6% or 0.3% of all hospitalizations), skin lesions were not related to an infectious disease. The most common discharge diagnoses were (Figure 2) cellulitis (various locations, typically lower limb) (*n* = 776, 33.9% of skin lesions), erysipelas (*n* = 620, 27.1%), herpes zoster (with or without complications) (*n* = 287, 12.5%), chickenpox (mostly with, sporadically without complications) (*n* = 177, 7.7%) and meningococcal infections (*n* = 61, 2.7%). There were 50 patients (2.2% of all skin lesions) in whom only a rather “vague” diagnosis of “unspecified viral infection characterized by skin and mucous membrane lesions” (ICD-10 code B09) was the leading discharge diagnosis (Figure 2). Individual non-infectious skin lesions such as erythema multiforme or nodosum, dermatitis due to substances taken internally (mostly drugs), contact allergic or non-allergic dermatitis and ulcer of the lower limb were sporadic and the only conditions observed in more than just a few patients (Figure 2).

The age of hospitalized patients ranged from <1 to 101 years (Table 3). Patients with infectious lesions appeared somewhat older than those with non-infections lesions, but age varied in respect to individual diagnoses, since some of the conditions are characteristic for children and younger adults (e.g., chickenpox, meningococcal infections), while other typically affect older people (e.g., cellulitis, erysipelas, herpes zoster) (Table 3). The median length of hospital stay was consistently 8–10 days, which was a bit shorter in patients hospitalized for (complicated) chickenpox (Table 3). 

## 4. Discussion

There are a few studies documenting the profile of patients with skin lesions in emergency departments (ED), all of which report an impressive percentage (2.5–8%) of all visits to the ED as being due to skin complaints [27,28,29,30,31,32,33,34,35,36,37,38]. 

In our infectious diseases ED, the profile of patients is different than in "classical" EDs that are mostly focused on internal medicine, surgical or neurological diseases. Patients with rash, with or without fever, as well as those with skin and soft tissue infections are quite common in our practice and according to our data, they make up almost 15% of all outpatients, which is higher than in other previously mentioned studies. A possible reason could be the fact that skin lesions are routinely ascribed an infectious etiology, especially when associated with constitutional symptoms.

The proportion of patients hospitalized due to skin lesions is high in our setting (13.1% of all hospitalizations). Among those, 13.1% and 61% are due to skin and soft tissue infections such as erysipelas and cellulitis, respectively. This is consistent with the overall increase in the incidence of skin and soft tissue infections, especially in populations over 65 years of age [9,39,40]. The median age of hospitalized patients with erysipelas and cellulitis in our study was 64 and 66 years, respectively. 

The highest median age was recorded in patients hospitalized due to herpes zoster (73 years), the incidence of which has also been increasing worldwide [41,42]. Age is the most important risk factor for the development of herpes zoster with a dramatic increase in the incidence rate after 50 years of age [43].

The incidence of life-threatening infectious diseases presenting with skin lesions (meningococcal infection, toxic shock syndrome, necrotizing fasciitis and staphylococcal scalded skin syndrome) was low in both outpatients (*n* = 37, 0.03%) and inpatients (*n* = 82, 0.5%). The number of patients diagnosed with meningococcal diseases, toxic shock syndrome and staphylococcal scalded skin syndrome was lower in ambulatory settings (*n* = 61, *n* = 10, *n* = 11, respectively) than in hospital settings (*n* = 23, *n* = 6, *n* = 8, respectively), which indicates that not all were recognized in the ED, but the diagnoses were established during hospitalization.

To date, the information we aim to present (the proportion of infectious, non-infectious and undiagnosed skin lesions) was also found in four published studies [16,44,45,46] but with a few differences, as shown in Table 4:

(1) Study population

In their study, Goodyear et al. [44] included only children, Tabak et al. [46] included only adults and Drago et al. [16,45] included both adults and children. Our study included adults and children as well.

(2) Inclusion criteria

Goodyear et al. [44] included children with acute erythematous rash with fever, but children with recognizable rashes such as classical measles and chickenpox were excluded. Drago et al. [16,45] included adults and children with clinical features which differ from classical exanthems. Finally, Tabak et al. [46] studied adult patients with fever and rash. We included all patients with infectious diseases that included skin lesions and those with common non-infectious skin lesions.

(3) Aim

Goodyear et al. [44] aimed to establish the etiology of non-classical erythematous rashes with fever in the pediatric population. Drago et al. [16,45] aimed to investigate whether morphology, other clinical features, laboratory results and other characteristics can help to determine the etiology of atypical exanthems. Tabak et al. [46] aimed to determine the etiology of fever and rash in adult patients. Our aim was to evaluate the quantity and type of skin lesions among outpatients and inpatients in order to assess disease burden and physicians’ experience in diagnosing skin lesions.

(4) Study design

Both Goodyear et al. [44] and Drago et al. [16,45] provided laboratory testing together with routine procedures (patient history and physical examination) in order to establish the etiology of exanthems. Tabak et al. [46] performed routine procedures and necessary tests according to suspected diagnoses. In our study, ICD codes were used to detect infectious disease patients with skin lesions, non-infectious skin lesions and undiagnosed skin lesions.

Although there is a significant difference between these four studies and ours, they were the only ones that used the term “undiagnosed” skin lesions/rashes in their analysis. Even though the results are not comparable with our own, due to the differences in study design and patient characteristics, the proportion of undiagnosed skin lesions in our study was smaller than Goodyear et al. [44] and Drago et al. [45] reported, as it only included patients with atypical exanthems. This difference is possibly due to the exclusion of patients with typical, recognizable rashes and the presence of more challenging patients. The proportion of undiagnosed skin lesions was higher than reported by Tabak et al. [46] in a study that included all adult patients with fever and rash. Finally, the proportion of undiagnosed skin lesions was similar to Drago et al. [16], even though it only included patients with atypical exanthems.

As dermatological disorders are quite commonly seen by almost all clinicians in their clinical practice, it seems logical to try to evaluate their ability in diagnosing skin conditions. Our clinicians are not the only ones commonly faced with diagnostic failures. Onsoi et al. [47] reported a large percentage (48.2%) of misdiagnoses of common cutaneous diseases in pediatric population. 

Antic et al. [25] reported that their internists recognized 51.1% of the cutaneous manifestations during examinations and 49% when presented with slides showing common dermatoses.

Because of the large number of illnesses that can manifest with different types of skin lesions, it is unreasonable to expect physicians to recognize all that comes into consideration while establishing a differential diagnosis. Working in pairs with more experienced colleagues during the learning period and practicing clinical image-based teaching as a supplement to the patient-based clinical teaching is the approach we suggest. Additionally, residents in infectious diseases should be exposed to dermatology training during residency, as it is currently not a required component of their training program. In addition, consultations with dermatologists are highly recommended in certain cases, especially if the physicians handling them are not routinely exposed to patients presenting with skin changes. This was already proven to be efficient in the study by Zhang et al. [48]. In the future, we might hope for a computational neural network to be developed in order to help with diagnosing skin lesions consistent with infectious diseases, as such a tool already exists for skin cancer [49,50].

The limitations of our study, listed below, arise from the use of ICD codes and bias introduced by potential misclassification and coding errors:

(1) It is possible that the number of patients with skin lesions is underreported due to the exclusion of diagnoses that include skin lesions as a non-compulsory part of the clinical presentation. These diagnoses include leptospirosis, bartonellosis, rotavirus infections, dengue fever, infections caused by mycoplasma pneumoniae, human immunodeficiency virus infection, Epstein–Barr virus infections, toxoplasmosis, malaria, etc.
(2) It is possible that there was a greater number of hospitalized patients with skin lesions that were not included in this study, as the skin lesions they may have presented with were not the cause of hospitalization or part of the primary diagnosis.
(3) The proportion of skin lesions classified as unspecified (R21 and R22) might be higher than reported due to individual classification preferences of clinicians. An example of such a preference is the fact that an unspecified cause can be also reported under L30 (Other dermatitis).
(4) Not all diagnoses could be classified unanimously as infectious or unrelated to infections. One such diagnosis is erythema nodosum (L52), which can occur secondary to a wide variety of conditions; however, an infection (primarily streptococcal) is the most commonly identified etiology [51].
(5) The reasons for using codes for unspecified skin lesions (R21, R22) can be multiple: (a) a skin lesion not being recognized; (b) uncertainty in the diagnosis—the coding system cannot determine the physician’s confidence in certain diagnoses; (c) a lack of code to accurately describe the condition. It is possible that not all skin lesions classified as “undiagnosed” were unrecognized.

Few studies have addressed the diagnosis of exanthems in adult and pediatric populations together with the proportion of infectious, non-infectious and undiagnosed exanthems as well as proportion of skin lesions caused by infectious agents among patients in emergency department. To date, no other study included all types of skin lesions that are infectious in etiology (mostly rashes with or without fever and local skin lesions such as skin and soft tissue infections) among patients visiting infectious disease settings including inpatients and outpatients. That is why, to our knowledge, our study is unique. 

## 5. Conclusions

The presented data suggest that the burden of diseases presenting with skin lesions in everyday infectious disease practice is large, especially in a specific setting that includes an emergency department and a hospital department for infectious diseases, and that the overwhelming number of undiagnosed outpatients implies the need for further education in this area. 

## Figures and Tables

**Table 1:** List of infectious diseases/ICD-10 codes searched.

**Bacterial diseases** A25 Rat-bite fevers	B35.0 Tinea barbae and tinea capitis B35.1 Tinea unguium
A26 Erysipeloid	B35.3 Tinea pedis
A31.1 Cutaneous mycobacterial infection	B35.4 Tinea corporis
A32.0 Cutaneous listeriosis	B35.5 Tinea imbricata
A36.3 Cutaneous diphtheria	B35.6 Tinea inguinalis (Tinea cruris)
A39 Meningococcal infection	B35.8 Other dermatophytoses
A43.1 Cutaneous nocardiosis	B35.9 Dermatophytosis, unspecified
A44.1 Cutaneous and mucocutaneous bartonellosis	B36 Other superficial mycoses
A46 Erysipelas	B36.0 Pityriasis versicolor
A48.3 Toxic shock syndrome	B36.1 Tinea nigra
A51.3 Secondary syphilis of skin and mucous membranes	B36.2 White piedra
A69.2 Lyme disease	B36.3 Black piedra
A75 Typhus fever	B36.8 Other specified superficial mycoses
A77.1 Spotted fever due to Rickettsia conorii	B36.9 Superficial mycosis, unspecified
A79.1 Rickettsial pox due to Rickettsia akari	B37.2 Candidiasis of skin and nail
**Viral diseases**	B38.3 Cutaneous coccidioidomycosis
A88.0 Enteroviral exanthematous fever (Boston exanthem)	B40.3 Cutaneous blastomycosis
A91 Dengue hemorrhagic fever	B43.0 Cutaneous chromomycosis
A92.0 Chikungunya virus disease	B45.2 Cutaneous cryptococcosis
B00 Herpes viral (herpes simplex) infections	B46.3 Cutaneous mucormycosis
B01 Varicella (chickenpox)	Parasitic diseases
B02 Zoster (herpes zoster)	B55.1 Cutaneous leishmaniasis
B03 Smallpox	B55.2 Mucocutaneous leishmaniasis
B04 Monkeypox	B65.3 Cercarial dermatitis
B05 Measles	B72 Dracunculiasis
B06 Rubella	B74 Filariasis
B07 Viral warts	B78.1 Cutaneous strongyloidiasis
B08 Other viral infections characterized by skin and mucous membrane lesions, not elsewhere classified	B85 Pediculosis and phthiriasis
B08.0 Other orthopoxvirus infections	B86 Scabies
B08.1 Molluscum contagiosum	B88.0 Other acariasis
B08.2 Exanthema subitum (sixth disease)	**Diseases caused by infectious agent**
B08.3 Erythema infectiosum (fifth disease)	L00 Staphylococcal scalded skin syndrome
B08.4 Enteroviral vesicular stomatitis with exanthem	L01 Impetigo
B08.5 Enteroviral vesicular pharyngitis	L02 Cutaneous abscess, furuncle and carbuncle
B08.8 Other specified viral infections characterized by skin and mucous membrane lesions	L03 Cellulitis
B09 Unspecified viral infection characterized by skin and mucous membrane lesions	L08 Other local infections of skin and subcutaneous tissue
**Fungal diseases**	L30.3 Infective dermatitis
B35 Dermatophytosis	L44.4 Infantile papular acrodermatitis (Giannotti–Crosti)

**Table 2:** List of non-infectious or non-specific skin lesions/ICD-10 codes searched.

L10 Pemphigus
L12 Pemphigoid
L13 Other bullous disorders
L20 Atopic dermatitis
L21 Seborrhoeic dermatitis
L22 Diaper [napkin] dermatitis
L23 Allergic contact dermatitis
L24 Irritant contact dermatitis
L25 Unspecified contact dermatitis
L26 Exfoliative dermatitis
L27 Dermatitis due to substances taken internally
L29 Pruritus
L30 Other dermatitis (except L30.3)
L40 Psoriasis
L41 Parapsoriasis
L42 Pityriasis rosea
L43 Lichen planus
L50 Urticaria
L51 Erythema multiforme
L52 Erythema nodosum
L53 Other erythematous conditions
L54 Erythema in diseases classified elsewhere
L55 Sunburn
L56 Other acute skin changes due to ultraviolet radiation
L70 Acne
L71 Rosacea
L73 Other follicular disorders
L84 Corns and callosities
L85 Other epidermal thickening
L88 Pyoderma gangrenosum
L89 Decubitus ulcer and pressure area
L90 Atrophic disorders of skin
L91 Hypertrophic disorders of skin
L92 Granulomatous disorders of skin and subcutaneous tissue
L93 Lupus erythematosus
L94 Other localized connective tissue disorders
L95 Vasculitis limited to skin, not elsewhere classified
L97 Ulcer of lower limb, not elsewhere classified
L98 Other disorders of skin and subcutaneous tissue, not elsewhere classified
R21 Rash and other nonspecific skin eruption
R22 Localized swelling, mass and lump of skin and subcutaneous tissue

**Table 3:** Age, gender and length of hospital stay overall and across most common diagnoses. Data are median (quartiles, range) or count (percent).

Diagnosis	*n*	Age (Years)	Men	Hospital Stay (Days)
All	2287	59 (34–74; <1–101)	1119 (48.9)	8 (6–11; 1–119)
All infectious	2228	59 (34–74; <1–101)	1097 (49.2)	8 (6–11; 1–119)
All non-infectious	59	44 (15–60; <1–90)	22 (37.3)	8 (5–14; 1–40)
Cellulitis	776	66 (51–76; <1–101)	355 (45.8)	9 (7–12; 1–33)
Erysipelas	620	64 (54–75; 2–94)	316 (51.0)	8 (6–10; 1–44)
Herpes zoster	287	73 (58–79; 8–97)	135 (47.0)	8 (6–11; 2–60)
Chickenpox	177	6 (2–28; <1–69)	100 (56.5)	5 (3–7; 1–23)
Meningococcal infection	61	2 (<1–15; <1–69)	31 (50.8)	10 (8–13; 1–49)
Erythema multiforme/nodosum	24	44 (29–55; 8–72)	9 (37.5)	8 (5–12; 3–22)

**Table 4:** Comparison of similar studies that reported undiagnosed skin rashes.

Authors	Year of Publication	No. of Patients	Characteristics of Patients	Inclusion Criteria	Undiagnosed
Goodyear HM, Laidler PW, Price EH, Kenny PA, Harper JI	1991	100	Children	Acute erythematous rash and a febrile illness of short duration Patients with recognizable rashes were excluded	*n*= 35, 35%
Drago F, Rampini P, Rampini E, Rebora A	2002	112	Children and adults	Rash other than classical exanthems	*n* = 36, 32%
Tabak F, Murtezaoglu A, Tabak O, Ozaras R, Mete B, Kutlubay Z, Mert A, Ozturk R	2012	100	Adults	Rash and fever	*n* = 10, 10%
Drago F, Paolino S, Rebora A, Broccolo F, Drago F, Cardo P, Parodi A	2012	260	Children and adults	Atypical exanthems other than classical exanthems	*n* = 59, 23%
Skuhala T, Trkulja V, Rimac M, Dragobratović A, Desnica B		21,114	Children and adults	All types of skin lesions	*n*= 4478, 21.1%

## Data Availability

We used institutional data that is not available for sharing.
